# Bridging Offline Functional Model Carrying Aging-Specific Growth Rate Information and Recombinant Protein Expression: Entropic Extension of Akaike Information Criterion

**DOI:** 10.3390/e23081057

**Published:** 2021-08-16

**Authors:** Renaldas Urniezius, Benas Kemesis, Rimvydas Simutis

**Affiliations:** Department of Automation, Kaunas University of Technology, LT-51367 Kaunas, Lithuania

**Keywords:** microbial cultivation, specific growth rate, oxygen uptake rate, functional model, model selection, recombinant protein concentration, target product

## Abstract

This study presents a mathematical model of recombinant protein expression, including its development, selection, and fitting results based on seventy fed-batch cultivation experiments from two independent biopharmaceutical sites. To resolve the overfitting feature of the Akaike information criterion, we proposed an entropic extension, which behaves asymptotically like the classical criteria. Estimation of recombinant protein concentration was performed with pseudo-global optimization processes while processing offline recombinant protein concentration samples. We show that functional models including the average age of the cells and the specific growth at induction or the start of product biosynthesis are the best descriptors for datasets. We also proposed introducing a tuning coefficient that would force the modified Akaike information criterion to avoid overfitting when the designer requires fewer model parameters. We expect that a lower number of coefficients would allow the efficient maximization of target microbial products in the upstream section of contract development and manufacturing organization services in the future. Experimental model fitting was accomplished simultaneously for 46 experiments at the first site and 24 fed-batch experiments at the second site. Both locations contained 196 and 131 protein samples, thus giving a total of 327 target product concentration samples derived from the bioreactor medium.

## 1. Introduction

Controlling and observing industrial biotechnology processes is a challenging task for bioengineers. The main problems are collecting accurate information regarding the state of the process and its quality. The industry demands the process be as productive as possible, which also contributes to the task’s difficulty. Overcoming these challenges requires high-quality and reliable process data. With concrete and quality data, easier process controllability and higher result repeatability are attainable. Unfortunately, the industry still lacks accurate and real-time measurements, especially for the main focus of almost all industrial cell cultivation processes—synthesized target product concentration. Sampled, time-delayed measurements with additional instruments and time-consuming analyses remain the most common way to determine the product concentration throughout cultivations. In large-scale processes, this problem becomes more acute, with additional hardware costs and the increased possibility of errors. Therefore, the realization and implementation of software sensors that can measure and predict indirect quantities using information collected throughout the process has become more prominent [1,2,3,4,5].

Target product concentration estimation in specific cultivations uses soft sensors that consist of various mathematical models [6]. These range from traditional mechanistic and empirical models to hybrid models, which have become increasingly prevalent for solving the estimation task. The conventional model’s classical shape requires elaboration and the tuning of its parameters to achieve satisfactory results [7]. Nevertheless, traditional mathematical models remain the fundamental basis of the software sensor, and in some instances, they are the most appropriate way to estimate process variables [8].

The use of traditional models for product estimation is seen in cultivations of *P. chrysogenum* for penicillin concentration [9], recombinant *E. coli* for protein concentration [10,11,12], and yeast fermentations for ethanol concentration [13]. Among the mechanistic unstructured models, the most popular approach is the extended Kalman filter [14,15]. However, the accuracy of the EKF and its results are closely related to the accuracy of the mathematical model, and may also suffer from convergence problems [16]. Nonetheless, EKF has considerable robustness to changes of initial process conditions, and has proven successful when applied in *S. cerevisiae* cultivations [6,17].

Applying traditional mathematical models to nonlinear and multidimensional systems may result in numerous errors due to the low flexibility of simple-structure differential equations. Therefore, researchers frequently choose an empirical model as an alternative approach that does not require detailed description of the process, but rather quantitative and qualitative data of the bioprocess. Among these data-driven models, the most successful and commonly applied are ANN (artificial neural networks), PLS (partial least squares), and PCA (principal component analysis)-based soft sensors. The latter, combined with spectroscopy, has been proven to provide satisfactory results in product estimation [18,19]. Meanwhile, ANNs have become crucial to hybrid models for product and state estimation [10,20]. The use of ANN is prominent not only as an alternative to describing complex parts of the processes, but also as a combination with additional off-gas analysis or spectroscopy data [21,22]. However, using such supplementary equipment for data gathering increases the process cost while also requiring added algorithms to compensate for the possible drifts in the gas sensors or data filtering from spectroscopy. Additionally, the estimation becomes time-delayed when taking samples periodically. Generally speaking, ANN-based software sensors, compared with traditional mathematical models, achieve more satisfactory results and require less development time [10,23].

A quick overview of the different techniques employed for specific product estimation can be seen in Table 1.

Our study aims to employ and expand the Luedeking–Piret model [25], and present an extension of the protein product estimation model based on gathered offline data. This paper improves the previous functional model by adding cell age and extensive model fitting analysis. The purpose of the proposed mathematical model is not to descriptively define the bioprocess, but instead to identify the correct state variables and their interrelationships that maximize synthesized product content.

Section 2: Materials and Methods describes the test object, processes, and operating conditions. Section 3: Proposed Extension of Akaike Information Criterion presents the modified Akaike criterion for model fitting with the addition of a tuning coefficient. Section 4: Combined Model Representing Multiple Hypothesis overviews previous similar maximal production rate expressions and proposes an improved model for target protein fitting. Section 5: System Identification and Parameter Estimation presents the model’s parameter identification methods and the use of cells ages. Section 6: Model Selection Based on Experimental Model Calibration compares the different models presented. Section 7: Discussion and Conclusions presents final remarks about the results and model fitting.

## 2. Materials and Methods

### 2.1. Cell Strains

The experimental object of this work was recombinant *E. coli* cells tested at two independent biopharmaceutical sites. The experimental data originate from cultivations of two different cell strains. The first cell strain was *E. coli* (BL21(DE3) pLysS (Site 1), and the second was *E. coli* BL21 (DE3) pET21-IFN-alfa-5 (Site 2). The synthesized product appeared in soluble and insoluble forms at both sites. The *E. coli* BL21 (DE3) target product was insoluble protein and inclusion bodies. The product’s expression was dependent on the T7 promoter, with one millimole of isopropyl-D-1-thiogalactopyranoside (IPTG).

### 2.2. Medium

For Site 1, the cultivation medium throughout the experiments consisted of Na_2_SO_4_, 2.0 g/L; (NH_4_)_2_SO_4_, 2.46 g/L; NH_4_Cl, 0.5 g/L; K_2_HPO_4_, 14.6 g/L; NaH_2_PO_4_ × H_2_O, 3.6 g/L; (NH_4_)_2_-H–citrate, 1.0 g/L; MgSO_4_ × 7H_2_O, 1.2 g/L; trace element solution, 2 mL/L [26].

For Site 2, the cultivations were based on a minimal mineral medium, consisting of 46.55 g KH_2_PO_4_, 14 g (NH_4_)_2_HPO_4_, 5.6 g C_6_H_8_O_7_.H_2_O, 3 mL of concentrated antifoam, 35 g H_14_MgO_11_S, and 105 g D (+) glucose monohydrate.

### 2.3. Cultivation Conditions

Table 2 presents the different cell cultivation conditions for both of the cell strains at both sites.

### 2.4. Target Protein Analysis

The analytical method of determining the amount of target protein was SDS-PAGE (sodium dodecyl sulfate–polyacrylamide gel) electrophoresis. The final measurement of the target protein consists of a sequence of the following actions. Firstly, 200 g of wet biomass was dissolved in 1 mL of solution and mixed for 30 min. Then, to measure the total protein concentration, SDS-PAGE electrophoresis was performed on 200 μL of the suspension sample. The remainder of the suspension was mixed with SDS (sodium dodecyl sulfate) buffer to dissolve all proteins and centrifuged for 15 min at 4 °C with 20,000 G force. Determining the soluble protein concentration required another SDS-PAGE electrophoresis with a sample of 200 µL. The leftover supernatant was discarded and replaced with 1 mL of water, then mixed and centrifuged. Finally, decanting the supernatant and mixing it for approximately 12 h with the addition of 1 mL of solubilization buffer (8 M urea; 50 mM, pH 8.0 Tris base) allowed for measurements of insoluble protein (inclusion bodies) concentration via SDS-PAGE electrophoresis.

## 3. Proposed Extension of Akaike Information Criterion

The classical form of the Akaike information criterion allows for selecting an informative set of parameters with an inevitable trade-off concerning the model’s fitting uncertainty [27]. Let *n* be the number of observation samples, *k* the number of model parameters, and *MSE* the mean squared error of the residuals. Then, the Akaike measure is

(1) AIC(k,n)=nln(MSE)+2·k.

An alternative is the Bayesian information criterion, or BIC, which contains variance $\sigma^{2}$ of errors instead

(2) $$BIC\left(k,n\right)=n\ln\left(\sigma^{2}\right)+2\cdot k.$$

One of the drawbacks of both *BIC* and *AIC* is that these criteria are designed to not have a tuning coefficient for minimizing the number of parameters to be used without changing the shape of the likelihood distributions. Another consideration is a tuning coefficient that would involve some theoretic asymptotic maximum number of parameters. In reality, the log-likelihood part of the criterion might not necessarily be related to the average characteristics, but they may also be cumulative characteristics based on the sum of squared residuals, RSS. This amount divided by the degree of freedom *n* recovers *MSE* and presents the average discrepancy between the readings $y\left(t_{i}\right)$ observed at time $t_{i}$ and the value estimated by the model $f\left(t_{i},k\right)$. Such cumulative discrepancy depends on the number of observations $n_{i}$, and has the form of

(3) $$RSS\left(k,n_{i}\right)=\sum_{i=1}^{n_{i}}{\left(y\left(t_{i}\right)-f\left(t_{i},k\right)\right)}^{2}=\sum_{i=1}^{n_{i}}{\left(y_{i}-f_{i}\left(k\right)\right)}^{2}.$$

Therefore, we suggest two entropic criteria for prospective model selection, which have a tuning coefficient $k_{\max}$, a likelihood $RSS\equiv RSS\left(k,n_{i}\right)$, and a maximum likelihood $RSS_{\max}\equiv RSS_{\max}\left(n_{i}\right)=\underset{k\to 0}{\lim}RSS\left(k,n_{i}\right)$, yielding

(4) $$S_{A}\equiv S_{A}\left(k_{\max},k,n_{i}\right)=\;\left(k_{\max}-k\right)\cdot RSS\ln RSS+k\cdot \left(RSS_{\max}-RSS\right)\ln\left(RSS_{\max}-RSS\right).$$

The other information measure, *S*, in the entropic representation, which can serve equally well, is

(5) $$S_{B}\equiv S_{B}\left(k_{\max},k,n_{i}\right)=\left(k_{\max}-k\right)\cdot RSS\cdot \ln RSS+k\cdot RSS_{\max}\cdot \ln\frac{RSS_{\max}}{RSS}.$$

Then, one can determine $k_{AIC}$ and $k_{BIC}$, with which

(6) $$RSS\equiv RSS\left(k,n_{i}\right)=\sum_{i=1}^{n_{i}}{\left(y\left(t_{i}\right)-f\left(t_{i},k\right)\right)}^{2}=\sum_{i=1}^{n_{i}}{\left(y_{i}-f_{i}\left(k\right)\right)}^{2}.$$

This links to Equations (1) and (2). In other words,

(7) $$AIC\left(k,n_{i}\right)\sim \underset{k_{\max}\to k_{AIC}}{\lim}\ln\left(S\left(k,n_{i}\right)\right),$$

and

(8) $$BIC\left(k,n_{i}\right)\sim \underset{k_{\max}\to k_{BIC}}{\lim}\ln\left(S\left(k,n_{i}\right)\right).$$

The motivation for tuning $k_{\max}$ to a certain $k_{optimal}$ is the need to avoid overfitting with experimental data when a user applies raw *AIC* or *BIC* criteria with a likelihood in any probabilistic form. Furthermore, the practical expectation is that the criterion be as generic as possible, and the likelihood’s shape should not require modification. Consequently, an investigator must pick such a set of parameters that mean minimal effort is required to perform a trial when seeking rational bioprocess optimization. For example, only one or two cultivation protocol changes should be made to potentially and noticeably increase the overall total product, i.e., by more than 10 percent or so. It is expected that a biopharmaceutical manufacturer performs as few changes as possible. Simultaneously, the manufacturer must follow for maximal repeatability and standardization according to EU CE labeling, EU medical device (MDR)*,* and US Food and Drug Administration (FDA) regulations at good manufacturing practice (GMP) or GMP-compliant (cGMP) facilities. This is particularly true when service providers provision a CDMO (contract development and manufacturing organization) technology transfer. Therefore, the upstream developers have one or two protocol adaptations or parameters at their disposal for a single experimental iteration consisting of unique experimental development trials or minor online checks.

In this study, we propose generic forms of Equations (4) and (5) that can be used to select such a minimal set of parameters that both reach (the principle of parsimony [28]) and match (the principle of convex optimization [29]) the extremum state of the measure.

## 4. Combined Model Representing Hypothesis with Multiple Elements

The previous study [11] introduced an additional protein P(t) production yield γ parameter to extend the Luedeking–Piret model for fed-batch cultivations [25,30,31]. The model relied on the oxygen uptake rate (OUR) for biomass *X* estimation

(9) $$OUR\left(t\right)=\alpha \cdot X^{\prime }\left(t\right)+\beta \cdot X\left(t\right)+\gamma \left(t\right)\cdot P^{\prime }\left(t\right),$$

The addition of production yield γ, which represents the oxygen consumption yield for the protein synthesis rate, supplements the previous cell’s oxygen consumption parameters for biomass growth α and maintenance β. The expanded model achieved a pseudo-global estimation of synthesized protein and biomass concentration [29,32,33]. Such a procedure corresponds to pseudo-global offline model calibration. It was assumed that protein yield was a function of biomass concentration in a gray box model [34].

As shown in a previous work, protein productivity depends on *IPTG* (isopropyl-D-1-thiogalactopyranoside) and biomass concentrations at time of induction [29,35]. The latter had a significant impact on the model, such that the product formation parameter γ became a function of biomass concentration at time of induction. Then, the final estimator form became

(10) $$OUR\left(t\right)=\alpha \cdot X^{\prime }\left(t\right)+k_{\gamma }\cdot \left(X\left(t\right)-X_{ind}\right)\cdot \frac{dP\left(t\right)}{dt}$$

The expression of the product model is based on the assumption of the linear dependency of product synthesis on the specific growth rate (SGR) of biomass [36]

(11) $$\frac{dP_{X}}{dt}=q_{px}\left(\mu ,\;P_{X}\right)=P_{\max}\left(\mu ,\;X\right)-k_{t}\cdot P_{X},$$

where $q_{px}$ is the specific protein accumulation rate (U/g/h), *µ* the specific biomass growth rate (1/h), and $P_{X}\equiv P\left(t\right)/X\left(t\right)$ the specific protein activity (U/g), where the protein concentration is normalized by biomass concentration. Even though the previous study assumed that the maximum target protein formation rate was linked to the specific substrate consumption rate, the underlying idea is still the same in this study. Finally, the time constant $k_{t}$ was assumed to have a self-inhibiting effect [37].

Over the years, multiple researchers have studied how different process variables and parameters affect the model of $P_{\max}$. Table 3 presents significant historic parametric developments.

D. Levisauskas and others expressed the maximal production rate ($P_{\max}$) via the concept of active biomass [38,39]. This latter is assumed to be the part of the biomass that is responsible for specific product production. The average cell age identifies the active biomass ${\bar{Age}}_{i}\equiv \bar{Age}\left(t_{i}\right)$ at any time $t_{i}$ throughout the bioprocess. The expression of average cell age, including the initial biomass boundary condition, is

(12) $${\bar{Age}}_{i}=\frac{X_{0}\cdot t_{i}+\int_{0}^{t_{i}}\left(t_{i}-t_{j}\right)\cdot X^{\prime }\left(t_{j}\right)dt_{j}}{X_{i}},$$

where $X_{0}$ is initial biomass at time of inoculation to a bioreactor. If the latter is assumed to be negligible, $\;{\bar{Age}}_{i}$ takes the following form

(13) $${\bar{Age}}_{i}=\frac{\int_{0}^{t_{i}}\left(t_{i}-t_{j}\right)\cdot X^{\prime }\left(t_{j}\right)dt_{j}}{X_{i}}≅\;\frac{\sum_{j=0}^{i}\left(t_{i}-t_{j}\right)\frac{\Delta X\left(t_{j}\right)}{\Delta t_{j}}\cdot \Delta t_{j}}{X_{i}}=\;=\frac{\sum_{j=0}^{i}\left(t_{i}-t_{j}\right)\Delta X\left(t_{j}\right)}{X_{i}}.$$

Equation (13) is the recovery of a particular case, shown in Equation (12), taken from D. Levisauskas and others’ research [38,39]. Assuming that $t_{j}\sim j\Delta t$, the maximal production rate $P_{\max}$ at time $t_{i}$ is

(14) $$P_{\max,1999}\left(t_{i}\right)=\frac{1}{X\left(t_{i}\right)}\overset{i}{\underset{j=1}{\sum }}\Delta X_{j}\cdot m\left(t_{i}-j\Delta t\right),$$

where $\Delta X_{j}$ is the growth of biomass throughout the *j*-th time interval, and *m* (0 < *m* < 1) is the relative activity ratio that introduces the linearly increasing and decreasing transient effect of the age. The parameter *m* is described by a trapezoid time function, which consists of four model parameters presumably related to each culture.

The most recent functional protein model [11] relies on the assumption that the maximal specific product concentration value is asymptotically dependent on *SGR*. However, the authors identified an apparent effect of *IPTG* injection on product synthesis through data analysis. Therefore, the functional model was expanded with the addition of biomass at induction time $X_{ind}$

(15) $$P_{\max,2019}\left(\mu ,\;X\right)=\mu \left(t\right)\cdot \left(k_{m0}+k_{m1}\cdot \left(X\left(t\right)-X_{ind}\right)\right)\;$$

where $k_{m0}$ and $k_{m1}$ are tuning parameters.

Other researchers [12] tried one more variation of the maximal product formation model

(16) Pmax,2003(μ)=μ(t)·kmkμ+μ(t)+μ2(t)kiμ. 

Such an approach was based on a rational assumption of what inhibits the maximal product formation rate. As far as we know, no efforts were made to test the different hypotheses of various methods with the same datasets originating from different sources. We propose a method of model selection using the principles of parsimony and convex optimization in this study. This is based on Equations (7) and (8).

With the combined approach of both product synthesis models, we include an expanded protein function model, where $P_{\max}\equiv P_{\max}\left(t\right)$ is the hypothesis of a mixture of linearly dependent competing models

(17) $$P_{\max,2013}=\sum_{l=1}^{n_{l}=24}P_{max,l},$$

where 24 model coefficients represent the parametric set of $k_{t},k_{0}\cdots k_{22}$, as defined in

(18) Pmax,2021=k0·μind+μ(k1(X(t)−Xind)+k3)+k2·μ·Age¯ind+k4·μ(k13+μind)+Xind(k6+k7·Age¯)+k8·Age¯+Age¯ind(k10+k11·μind)+k12·μind2+k16·μind·Age¯k20+Age¯+k17·μk19+μ+k18·μ·Age¯k21+Age¯+k22·μ·Age¯indk5+Age¯ind+k9·μk14+μ+μ2k15.

Here, $k_{t},k_{0}\cdots k_{22}$ are the optimization parameters of the model to be established. All of them contain zero values at the start of the convex search. The subset of linear terms represents the linear term of Equation (18), and some of them are the basis of Monod’s formulation theories [40,41]. The matches are depicted in Table 4.

The novelty of this study is the proposed average cell age at induction time $Age_{ind}$. As the researchers [38,39] did not study the recombinant bioprocess in their work, so far, the effect of IPTG injection has not been assessed. Based on the experimental data, we deduced that the average cell age and specific growth rate during the induction time are the most significant parameters to consider when creating a protein formation model.

## 5. System Identification and Parameter Estimation

### 5.1. Average Cell Age at the Induction

Historically, mathematical bioprocess models have considered only external state variables that affect product biosynthesis. For this reason, traditional models show frequent inconsistency when validating theoretical knowledge with empirical data. To improve the accuracy and applicability of the model, we considered variations in the physiological state of the microorganisms, including, but not limited to, their physical age, similarly to the developments made in the 1970s [42]. Consequently, we express the average cell age at induction time ($t_{ind}$) as

(19) $${\bar{Age}}_{ind}\equiv \bar{Age}\left(t_{ind}\right)≅\frac{X_{0}\cdot t_{ind}+\int_{0}^{t_{ind}}\left(t_{ind}-t_{j}\right)\cdot X^{\prime }\left(t_{j}\right)dt_{j}}{X_{ind}}.$$

The use of cell age relies on two main assumptions. The first is that the total biomass does not produce the specific product, only its physiologically active part. The second is that the activity of the biomass depends on its age. Therefore, through our modeling, we can predict that the cells produce the specific product throughout a particular period, during which there is an average cell age that would lead to maximal production. This also relates to induction, at which point the cells have already reached a certain age.

### 5.2. Model of Product Model Fitting

Following the presented changes, the previously described relative protein synthesis Equation (11) has a more general presentation

(20) $$\frac{dP_{X}}{dt}\equiv q_{px}\left(\mu ,\;P_{X}\right)=P_{\max}\left(\mu ,\;X,t\right)-k_{t}\cdot P_{X}$$

Furthermore, its integral form at time *t* becomes

(21) $$P_{X}\left(t\right)=\int_{t_{0}}^{t}P_{\max}\left(t^{*}\right)dt^{*}-k_{t}\cdot \int_{t_{0}}^{t}P_{X}\left(t^{*}\right)dt^{*},$$

where the integrals are the left-hand Riemann sum [11,43]. Finally, the protein model for pseudo-global offline fitting takes the form

(22) $$P_{i}=\frac{\left(\sum_{j=1}^{i}P_{\max,j}\cdot \Delta t_{j,j-1}-k_{t}\cdot \sum_{j=1}^{i-1}P_{X,j}\cdot \Delta t_{j,j-1}\right)\cdot X_{i}}{1+\Delta t_{i,i-1}\cdot k_{t}}.$$

In Equation (22), the discrete protein values define the variable $P_{X,i}\equiv P_{X}\left(t_{i}\right),$ where the sample observed at time *t* is indexed by *i*, and $i\in \left[1,n_{i}\right]$.

### 5.3. Pseudo-Global Offline Identification of Model Parameters

Before selection, each model requires pseudo-global parameter identification. The identification process of protein model fitting coefficients consists of the convex optimization method and the maximization of entropy [28,44,45]. Based on Bayesian analysis, the posterior distribution for the *i*-th offline sample is expressed as

(23) $$P_{\mathrm{posterior}}\left(P_{i}\right)\sim N\left(P_{i},\sigma_{P}^{2}\right),$$

where $\sigma_{P}^{2}$ is the constant variance for every sampled prediction *i*. Similarly, the prior distribution has the following form

(24) $$P_{\mathrm{likelihood}}\left(P_{i}\right)\sim N\left(P_{i}^{y},\sigma_{P,i}^{2}\right),$$

where $P_{i}^{y}$ is the *i*-th observed value of product concentration with an individual variance $\sigma_{P,i}^{2}$. Having both distributions leads to a simplified form of relative entropy, which serves as a likelihood function for the posterior,

(25) $$L_{i}\equiv S_{i}\left(P_{\mathrm{posterior}},P_{\mathrm{likelihood}}\right)=-\frac{{\left(\langle P_{i}\rangle -P_{i}^{y}\right)}^{2}}{2\cdot \sigma_{P,i}^{2}}+c.$$

In a previous study, we neglected coefficient *c* in favor of a separate tuning coefficient $K_{\exp}\;\left(0\leq K_{\exp}\leq 2\right)$ [11,29]. The coefficient is implemented to adjust for trade-offs between the least squares and mean absolute percentage error approaches. Such a combination takes advantage of both criteria. With the addition of $K_{\exp}$, the expression of relative entropy becomes

(26) $$L_{i}=-\frac{{\left(\langle P_{i}\rangle -P_{i}^{y}\right)}^{2}\cdot \left(1-K_{\exp}\right)}{2\cdot P_{i}^{y,2}}-\frac{{\left(\langle P_{i}\rangle -P_{i}^{y}\right)}^{2}\cdot K_{\exp}}{2}.$$

The process of model fitting uses the former equation to identify the product model’s parameters. The use of convex optimization with parsimony assumptions allows the entropy measure to indicate local extremums and derive a sufficient computational processing time [28]. For simplicity, and given that the protein content did reach high concentrations, the $K_{\exp}$ was set to 2 in this study. Therefore, the residual sum of squares denotes the squared sum, which thus represents the likelihood in the ensuing text.

## 6. Model Selection Based on Experimental Model Calibration

We analyzed two datasets in this study, derived from different samples from two independent sites. The first repository consisted of 46 independent experiments and, in total, $n_{i,I}=196$ readings. The other dataset, from the second site, contained 24 unique biosyntheses and, in total, $n_{i,II}=131$ protein observations. To use a single RSS with $n_{i}=n_{i,I}+n_{i,II}$ in the same model selection routine, we picked a normalized form by reusing two sums of squared residuals ($RSS_{I}$ and $RSS_{II}$) for each site

(27) $$RSS=\frac{n_{i,II}\cdot RSS_{I}+n_{i,I}\cdot RSS_{II}}{n_{i}}.$$

This allowed for distributing the average variances of the estimates evenly over both sites’ repositories. After the maximization of Equation (26), a convex search of the data from previous studies gave the results shown in Table 5. To check for errors at the beginning of product synthesis, we added to the evaluation the criteria of mean absolute error (*MAE*).

(28) $$MAE=\frac{\sum_{i=1}^{n}\left|P_{i}-P_{i}^{y}\right|}{n}.$$

At first glance, according to the AIC in Table 5, the investigation from 2019 [11] improved on the studies from 1999 [38,39] and 2003 [12]. Then, the study of 2003 [12] improved upon the AIC of 1999 [11]. However, according to the MAE criterion, which is more relevant to product formation, the oldest assumption in the literature [38,39] is more powerful than the newer findings derived over 20 years later. Moreover, if the *AIC* were to be followed literally, the overfitting of the overall model would have been favored, as the last row of Table 5 demonstrates. Such an elaboration led us to further study the product formation model, and search for better ways of selecting a model with fewer parameters and which avoids overfitting by design.

First of all, there is a possible value for the maximum number of coefficients ($k_{\max}$) that asymptotically makes the entropic criteria work the same way as the original *AIC* and *BIC* measures. The maximization of correlation between *AIC* and $S_{A}$ (Equation (4)), and then $S_{B}$ (Equation (5)), generates corresponding $k_{\max}$ values $k_{AIC,A}$ and $k_{AIC,B}$, which are shown in Table 6.

Similarly, maximizing the linear relationship between *BIC* and $S_{A}$, and then $S_{B}$, provides the data for Table 7. We asymptotically tuned both *AIC* and *BIC* on the sum of correlations of 33 models, which together comprised a specific subset of Equation (18). We tried more reproductions with different assumptions in this study. However, those 33 representations comprising Equation (18) are the best set, according to our investigation experience. The maximal parametric complexity we tried was k≤6 in this study.

Table 6 and Table 7 both show that each entropic measure of *S* is a more generic quantity that can help restrict the number of expected state variables, thus helping with upstream *CDMO* development in the biopharmaceutical industry. Typically, two to four coefficients are preferred in optimal control routines, because the degree of freedom in Hamiltonians intensifies computational requirements. The main reason for this is that, frequently, Hamiltonians are solved numerically or using hybrid approaches, of which arithmetic processing still represents an extensive part. As such, we present experimental findings for a maximal number of model parameters of $k_{max}=k_{AIC}=k_{BIC}=450$, unless specifically stated otherwise.

Before proceeding with model selection, we must check the significance of the tuned model parameters individually. We select $k_{t}$ and two other coefficients with state variables and a significant history [11,12,38,39], which we found to be the best descriptors.

The specific growth rate at time of induction is the most significant parameter from a singleton analysis perspective, as Table 8 shows. This table offers two insights:

(a) There is significant doubt that $k_{t}$ belongs to the descriptor set;
(b) Even if the specific growth rate surpasses the average cell age, the significance of either is still relatively similar. Therefore, there is a high chance that both of them combine in a single nonlinear relationship that is proportional to the maximum product formation rate.

Such thinking led us to construct maximum product expression, as in Equation (18). We will use the maximum number of models assessed during our criterion asymptotic analysis, and set $k_{\max}=33$. The five best model equations that derive from Equation (18) are

(29) $$P_{\max}=k_{0}\cdot \left(\mu_{ind}-\mu_{ind}^{2}\right)+\frac{k_{16}\cdot \mu_{ind}\cdot \bar{Age}}{k_{20}+\bar{Age}}+k_{16}-k_{16}\cdot \mu \;\mathrm{and}\;k_{t}=0,$$

(30) $$P_{\max}=k_{0}\cdot \mu_{ind}+\frac{k_{16}\cdot \mu_{ind}\cdot \bar{Age}}{k_{20}+\bar{Age}}+k_{16}-k_{16}\cdot \mu \;\mathrm{and}\;k_{t}=0,$$

(31) $$P_{\max}=k_{0}\cdot \mu_{ind}+\frac{k_{16}\cdot \mu_{ind}\cdot \bar{Age}}{k_{20}+\bar{Age}}+k_{16}\;\mathrm{and}\;k_{t}=0,$$

(32) $$P_{\max}=\frac{k_{16}\cdot \mu_{ind}\cdot \bar{Age}}{k_{20}+\bar{Age}}+k_{16}\;\mathrm{and}\;k_{t}=0.$$

Table 9 depicts the parameter values of the models in Equations (29)–(32).

The second additive term, as used in Equations (29)–(32), and the first additive term, as used in Equation (32), is the Monod term, whose coefficients $k_{16}$ and $k_{20}$ carry a specific physiological meaning: the maximum specific target protein formation rate is the multiplication $k_{16}\cdot \mu_{ind}$; the denominator additive coefficient defines the average age at which the production formation rate (represented by term $k_{16}\cdot \mu_{ind}$) is halved. The perfect average age for inoculation is somewhere between 1.066 h and 1.3 h, at which point product formation has the highest theoretical rate of acceleration. It remains to be determined whether it is a coincidence that the minimum induction time was 1.14 h for the first site and 1.237 h for the second site.

As the mean absolute error is the smallest for the model with more variables in Equation (29), other maximal counts of model parameters remain to be verified. The asymptotic analysis using $k_{\max}=6$, which is the maximum number of tested parameters per experiment in this study, suggests the following five alternatives:

(33) $$P_{\max}=k_{0}\cdot \mu_{ind}\;\mathrm{and}\;k_{t}=0,$$

(34) $$P_{max}=k_{8}\cdot \bar{Age}\;\mathrm{and}\;k_{t}=0,$$

(35) $$P_{max}=\frac{k_{16}\cdot \mu_{ind}\cdot \bar{Age}}{k_{20}+\bar{Age}}+k_{16}-k_{16}\cdot \mu \;\mathrm{and}\;k_{t}=0,$$

(36) $$P_{max}=k_{0}\cdot \mu_{ind}\;\mathrm{and}\;k_{t}=0.447,$$

(37) $$P_{max}=k_{8}\cdot \bar{Age}\;\mathrm{and}\;k_{t}=2.059.$$

Table 10 shows another alternative set of coefficients, which verify that the average age has a more substantial effect at the start of product formation. Thus far, Equation (29) gives the best estimate of the total product.

There is still one model to consider, which can improve *MAE* to 0.424

(38) $$P_{\max,2021}=\mu \left(k_{1}\left(X\left(t\right)-X_{ind}\right)+k_{3}\right)\;\mathrm{and}\;k_{t}=-0.112,k_{1}=-0.00243,k_{3}=0.074.$$

However, this model’s *RSS* is poor, at 14.826. Further increasing the number of parameters starts to reduce the *MAE* due to overfitting.

## 7. Discussion and Conclusions

The results of the model selection and the application of enhanced *AIC* show two things:

(a) As regards rational, practical benefits, the proposed entropic measures can help with tuning the maximum count of the model parameters, thus helping devise standardized CDMO procedures for attaining higher product yields from biopharmaceutical efforts;
(b) Secondly, both average age and biomass growth values at time of induction, or in other words, at the very start of product synthesis, are crucial. Therefore, the combined model employing Monod structures is the best recommendation for maximizing the total product yield.

Similar to the Akaike information criterion, the Bayesian information criterion can also be viewed as a particular asymptotic enhancement of the entropic expansion of *AIC*. Such an approach avoids altering the likelihood or re-organization the experiments. Instead, it brings the benefit of adjustability in the maximum number of expected coefficients. Moreover, two entropic values are available for scientists to exploit: relative entropy and Shannon entropy. The experimental model fitting was performed simultaneously on 46 experiments at the first site and 24 fed-batch experiments at the second site. Both locations contained 196 and 131 protein samples, thus giving a total of 327 target product tests using the bioreactor medium.

Regarding the physiological characteristics of any aerobic microbial system, we witnessed that average cell age and the inhibition coefficient are both more relevant, and describe the model better, at the very beginning of product biosynthesis. At the same time, the specific growth rate improves upon the latter overall, when considering the total (recombinant target protein) expression at the end of the experiments.

## Figures and Tables

**Table 1:** Examples of different modeling techniques for product estimation.

Model Type	Model Structure	Comment	Product		Reference
			Soluble	Insoluble	
**Conventional** (based on balance equations)	Balance of production rate	Assessment of dilution and product concentration, hard to distinguish between estimation and prognostication	Penicillin V	-	[9]
	Balances of specific substrate uptake and growth rate	A hybrid model provides better results than a traditional one	Recombinant protein	-	[10]
	Balances of biomass, specific growth rate, production rates	-	-	Recombinant protein	[11]
	Balance of biomass, specific growth rate, and protein activity	Optimization for maximal protein using induction time and feed profiles	Recombinant protein		[12]
	Balance of biomass, pH, added ammonia	-	Ethanol	-	[13]
	Spectroscopy data analysis with EKF	-	Ethanol	-	[17]
**Empirical** (data driven)	Spectroscopy data analysis with PLS	-	Penicillin V		[18]
	Spectroscopy data analysis with PCA	-	-	Recombinant antibodies from mammalian cells	[19]
	Off-gas analysis with ANN	Gas sensors suffer from signal drift which requires additional compensation	-	Recombinant human blood coagulation factor VIII	[21]
**Hybrid**	ANNs for product formation rate and specific growth rate	-	Recombinant protein		[10]
	ANN for dissolved oxygen assessment	The assumption is valid only when the PID parameters for controlling the DO circuit are unchanged	Penicillin		[20]
	ANN with inputs of biomass, dilution rate, etc.	-	Ethanol		[23]
	Support vector regression for observations of oxygen undertake, carbon production, and base consumption rates	The presented model is for prediction, not for pseudo-global estimation	-	Recombinant protein	[24]

**Table 2:** The cultivation conditions of Site 1 and Site 2 cell strains.

Condition	Site 1	Site 2	Note
Bioreactor Volume	15 L	7 L	-
Cultivation Type	Fed-batch	Fed-batch	-
Temperature Setpoint	30 °C	37 °C	Both measured with a PT100 temperature sensor
DO Setpoint	30%	20%	Both measured with an Ingold DO probe (Mettler Toledo)
pH Setpoint	7	6.8	Both kept constant using a PID controller with the addition of NaOH
Stirrer Setpoint Range	100–1400 RPM	800–1200 RPM	-
Airflow	0.3–15 L/min	1.75–3.75 L/min	Pure oxygen flow was provided to bioreactors at a range from 0 to 7.5 L/min to increase the oxygen transfer rate
Maximum average cell age at induction, hours	3.105	2.985	-
Minimum average cell age at induction, hours	1.14	1.237	-
Off-gas Tracking	Concentrations of O_2_ and CO_2_	Concentration of O_2_	Measured with a paramagnetic oxygen sensor (Maihak Oxor 610) during Site 1 cultivations and with BlueSens gas analyzer (BCpreFerm, BlueSens, Herten, Germany) during Site 2 cultivations.

**Table 3:** Hypothetical dependencies of the maximum specific product formation rate.

$P_{\max}\;\mathrm{Arguments}$	State Variables	Reference(s)	Equation
$a_{1},a_{2},a_{3},a_{4}$	μ(t),X(t) or $\bar{Age}\left(t\right)$	1999, [38,39]	(14)
$X_{ind},k_{m0},k_{m1}$	μ(t), X(t)	2019, [11]	(15)
$k_{m0},k_{\mu },k_{i\mu }$	μ(t)	2003, [12]	(16)

**Table 4:** Product formation rate dependencies that are part of Equation (18).

$P_{max}\;\mathrm{Arguments}$	State Variables	Model Selection Arguments in This Study	Reference(s)
$a_{1},a_{2},a_{3},a_{4}$	$\bar{Age}\left(t\right)$	$k_{8}$	1999, [38,39]
$X_{ind},k_{m0},k_{m1}$	μ(t), X(t)	$k_{1},k_{3}$	2019, [11]
$k_{m0},k_{\mu },k_{i\mu }$	μ(t),	$k_{9},k_{14},k_{15}$	2003, [12]
$Age_{ind},\mu_{ind}$, etc.	$\mu \left(t\right),\;X\left(t\right),\bar{Age}\left(t\right)$	$k_{t},k_{0}\cdots k_{22}$	2021/this study

**Table 5:** Product’s *AIC*, *RSS*, and *MAE* statistics in each historical study.

*AIC*	*RSS*	*MAE*	*k*	Model Selection Arguments	Reference(s)
−967.01	16.79	0.393	2	$k_{t}≅2.06,$ $k_{8}≅0.01176;$	1999 [38,39]
−1005.6	14.83	0.424	3	$k_{t}≅-0.112,$ $k_{1}≅-0.00243,$ $k_{3}≅0.074;$	2019 [11]
−977.17	16.07	0.442	4	$k_{t}≅0.321,$ $k_{9}≅0.01193,$ $k_{14}≅-0.000473,$ $k_{15}≅0.1677;$	2003 [12]
−1488.16	3.15	0.249	24	$k_{t}≅0.209,k_{0}\cdots k_{22};$	Full overfit with Equation (18)

**Table 6:** Product’s *AIC* as an asymptotic assessment of entropic measures $S_{A}$ and $S_{B}$.

*AIC*	$\ln S_{A}$ $k_{AIC,A}\to 830$	$\ln S_{B}$ $k_{AIC,B}\to 450$	Reference(s)
−967.01	10.583	9.968	1999 [38,39]
−1005.6	10.419	9.802	2019 [11]
−977.17	10.53	9.9117	2003 [12]

**Table 7:** Product’s *BIC* as an asymptotic assessment of entropic measures $S_{A}$ and $S_{B}$.

*BIC*	$\ln S_{A}$ $k_{BIC,A}\to 300$	$\ln S_{B}$ $k_{BIC,B}\to 172$	Reference(s)
−959.430	9.573	9.008	1999 [38,39]
−994.228	9.417	8.848	2019 [11]
−962.013	9.529	8.956	2003 [12]

**Table 8:** Significance test for single parameters.

Parameter and Its Value	State Variable or Argument	*AIC*	*BIC*	$\ln S_{AIC,A}$	$\ln S_{AIC,B}\;$
$k_{t}≅53.9$	$P_{X}\left(t\right)$	−591.28	−587.49	-	10.5
$k_{0}≅0.0159$	$\mu_{ind}$	−936.78	−932.99	9.145	9.138
$k_{8}≅0.001384$	$\bar{Age}\left(t\right)$	−905.04	−901.25	9.273	9.267

**Table 9:** Parameter values for the significance test at $k_{\max}=33$.

Equation	$k_{0}$	$k_{16}\;$	$k_{20}\;$	$\ln S_{AIC,A}$	*RSS*	*MAE*	*k*
(29)	−0.13	0.0232	−1.066	6.869	7.279	0.399	3
(30)	−0.0375	0.01148	−1.244	6.979	8.723	0.432	3
(31)	−0.0337	0.0098	−1.261	6.998	8.970	0.462	3
(32)	0	0.00298	−1.302	7.099	11.782	0.579	2

**Table 10:** Parameter values for the significance test with $k_{\max}=6$.

Equation	$k_{0}$	$k_{8}$	$k_{16}\;$	$k_{20}\;$	$\ln S_{AIC,A}$	*RSS*	*MAE*	*k*
(32)	0.0159	0	0	0	5.976	18.524	0.639	1
(34)	0	0.00138	0	0	6.047	20.412	0.497	1
(35)	0	0	0.00321	−1.298	6.054	11.857	0.577	2
(36)	0.0453	0	0	0	6.109	16.475	0.603	2
(37)	0	0.01176	0	0	6.114	16.785	0.393	2

## Data Availability

Data sharing does not apply to this article.
